# Robot-Assisted Laparoscopic Radical Prostatectomy in the Morbidly Obese Patient

**DOI:** 10.1155/2011/618623

**Published:** 2010-11-29

**Authors:** Jennifer Yates, Ravi Munver, Ihor Sawczuk

**Affiliations:** ^1^Department of Urology, Hackensack University Medical Center, 360 Essex Street, Hackensack, NJ 07601, USA; ^2^Touro University College of Medicine, Hackensack, NY 10027, USA; ^3^New Jersey Medical School, University of Medicine and Dentistry of New Jersey, Newark, NJ 07103, USA

## Abstract

*Introduction*. Obesity and prostate cancer are among the more common health issues affecting men in the United States. *Methods*. We retrospectively reviewed morbidly obese (BMI ≥ 40 kg/m^2^) patients undergoing RALP between 2004–2009 at our institution. Parameters including operative time, estimated blood loss, hospital stay, pathology, and complication rate were examined. *Results*. A total of 15 patients were included, with a mean BMI of 43 kg/m^2^. Mean preoperative PSA was 5.78 ng/dL, and Gleason score was 6.6. Mean operative time was 163 minutes, and mean estimated blood loss was 210 mL. The mean hospital stay was 1.3 days. Positive margins were noted in 2 (13%) patients, each with pT3 disease. There were no blood transfusions, open conversions, or Clavien Grade II or higher complications. *Conclusions*. In our experience, RALP is feasible in morbidly obese patients. We noted several challenges in this patient population which were overcome with modification of technique and experience.

## 1. Introduction

The prevalence of obesity in the United States has increased over the past few decades. Recently, the National Health and Nutrition Examination Survey (NHANES) reported an age-adjusted prevalence of obesity in 2007 and 2008 to be 33.8% in men and 35.5% in women [1]. While in past years the prevalence of obesity has increased dramatically, it appears that the rate of increase may be slowing. The World Health Organization (WHO) definition of obesity is based on body mass index (BMI), and includes Grade I (BMI between 30 and 34.9 kg/m^2^), Grade II (BMI between 35 kg/m^2^ and 39.9 kg/m^2^), and Grade III (BMI of 40 kg/m^2^ and higher). Obesity contributes to a number of health conditions, including diabetes mellitus, hypertension, osteoarthritis, and some malignancies [2]. In regards to prostate cancer, some data suggests that obese patients may have less favorable disease pathology [3–5].

Obesity can make any surgical procedure more challenging and increase the risks of morbidity. The introduction of robot-assisted laparoscopic radical prostatectomy (RALP) can help overcome some of these technical difficulties, but also introduces other procedural challenges. Several institutions have reported experience in the obese population with open radical retropubic prostatectomy (RRP), radical perineal prostatectomy (RPP), and minimally invasive radical prostatectomy (MIRP), including laparoscopic radical prostatectomy (LRP), and RALP [6–23]. Many of the patients included in these studies meet the criteria for Class I or Class II obesity, with a lower incidence of Class III obesity.

At our institution, patients with prostate cancer are not excluded from surgical management on the basis of obesity alone. We have subjectively found that RALP in the obese, and especially the morbidly obese patient, can be technically challenging. We sought to describe the objective outcomes of RALP in the morbidly obese patient, and to review the literature for outcomes of obese patients undergoing radical prostatectomy.

## 2. Methods

After obtaining institutional review board (IRB) approval, the records of patients undergoing RALP between 2004 and 2009 by 2 surgeons (RM, IS) at our institution were reviewed. Morbidly obese patients, that is, Class III obesity (BMI ≥ 40 kg/m^2^), were identified and included in the review. The medical records were reviewed for relevant demographic factors, clinical staging characteristics, operative time, estimated blood loss (EBL), length of hospital stay (LOS), blood transfusion rate, incidence and type of complication, and pathological characteristics. 

## 3. Results

A total of 15 patients with a mean BMI of 43 kg/m^2^ (range 40–53) and mean weight of 128.2 kg (range 93–173) were identified. Medical comorbidities included hyperlipidemia in 87%, diabetes mellitus in 27%, hypertension in 73%, and coronary artery disease in 20%. Mean preoperative prostate specific antigen (PSA) was 5.78 ng/dL and mean biopsy Gleason score was 6.6 (range 6–8). Mean operative time was 163 minutes (range 115–250) and mean estimated blood loss was 210 mL (range 50–400). Patients remained in the hospital on average 1.3 days (range 1-2) after surgery. Final pathology revealed pT2a (*N* = 3), pT2c (*N* = 8), pT3a (*N* = 3), and pT3b (*N* = 1). The pathologic Gleason score was 5 (*N* = 1), 6 (*N* = 6), 7 (*N* = 7), and 9 (*N* = 1). Mean prostate volume was 48.5 grams (range 37–62). Positive margins were noted in 2 (13%) of the patients, each of whom had pT3 disease. There were no blood transfusions, conversion to an open procedure, case abortions, or Clavien grade II or higher complications.

In comparison, 365 nonobese patients undergoing RALP during the same time period had a mean BMI of 27.8. In this group, mean operative time was 180.7 minutes (range 90–540) and mean estimated blood loss was 134 mL (range 25–500). The positive margin rate was 17.5%. Mean length of stay was 1.1 days. 

## 4. Discussion

The challenges of treating obese patients are not limited to the urologic field, and experience across all medical fields expands as the incidence of obesity rises. From an anesthesia standpoint, obese patients have a higher rate of medical comorbidities [16]. These patients can be more challenging for the anesthetist to manage during laparoscopic surgery, as they may demonstrate arterial oxygenation insufficiency and higher peak inspiratory pressures compared to non-obese patients [24]. Obese patients may also be more difficult to intubate due to altered neck or airway anatomy and may have increased risk of pulmonary aspiration due to higher gastric residual volumes. Vascular access can also be more challenging due to excess soft tissue [25]. In the early postoperative period, obese patients are at risk for pulmonary complications due to the higher incidence of obstructive sleep apnea (OSA) and the obesity hypoventilation syndrome. When combined with general anesthesia and narcotics, even mild cases of OSA can be dangerous. These patients should be closely monitored postoperatively to prevent serious pulmonary complications [25]. 

The challenges associated with obese surgical patients are documented in the general surgery literature. Dindo [26] reported outcomes of obese patients undergoing elective general surgery procedures, finding these patients demonstrated an increased need for blood transfusions and a slightly higher risk of wound infection. The bariatric surgery literature suggests that high-volume surgeons and medical centers, those most accustomed to treating the obese patient, have superior bariatric surgery outcomes [27, 28]. Both length of stay and mortality were decreased at one institution that reports high volumes of bariatric surgery [27]. 

Obesity as a factor in the surgical treatment of prostate cancer has several implications. First, the cost of treating the obese patient may be greater than treating a non-obese patient. Bolenz [29] compared the costs associated with RALP, RRP, and LRP in both obese and non-obese patients. The authors found that all individual cost domains were higher in the obese except for the room and bed cost. They suggest this is mostly due to the higher operating room service and anesthesia costs associated with longer operating times. When broken down by surgical technique, both LRP and RRP were associated with higher costs in the obese patient, but RALP showed no difference in cost when comparing obese and non-obese patients.

Oncologic outcomes in obese patients may be less favorable when compared to non-obese patients. Freedland [30] found a statistically significant association between patient obesity and positive surgical margins (PSMs) early in the cohort's experience with RRP. Later in the experience, there was a trend toward higher grade disease in obese patients. They conclude that while the association between obesity and PSM has decreased over time, the association between obesity and higher grade cancer and advanced stage has become stronger. In another review of patients undergoing RRP, Siddiqui et al. [3] found that obese patients had slightly higher rates of Gleason score 8–10 on both biopsy and pathology specimens. In addition, PSM's were more common in the obese patients. Body mass index did not affect biochemical recurrence-free survival, cancer-specific survival, or overall survival. In a series of patients undergoing RALP, Boorjian et al. [8] found that PSMs were more likely to be found posteriorly in the obese patients and at the apex in non-obese patients. Overweight and obese patients were also more likely to have pathologic stage T3 and T4 disease, higher grade disease, and higher volume tumors. While still controversial, it is possible that obesity can impact tumor biology, predisposing obese patients to more aggressive variants of prostate cancer.

The surgical outcomes of radical prostatectomy in the obese patient vary by the type of surgery and by series. Radical perineal prostatectomy (RPP) is believed by some to be advantageous in the obese, as the anterior abdominal panniculus and dorsal venous complex are avoided. Yang et al. [19] compared obese and non-obese patients undergoing RPP. Similar estimated blood loss, length of stay, operative times, and PSM's were noted, but obesity was associated with the risk of complications, especially lower extremity neuropraxia. In a group of morbidly obese patients (average BMI 39.3) undergoing RPP, Boczko reported no complications and no transfusions in seven patients [22]. 

Open radical retropubic prostatectomy in obese patients was described by Lindner [13]. While the incidence of perioperative complications and blood transfusions was similar, the authors found that EBL was higher in the obese group and operative times in the obese were longer. Obese patients experienced a similar length of hospital stay, PSM rate, and pathologic Gleason score and stage. Another series reported a higher rate of wound infection, urinary incontinence, and bladder neck contracture in obese patients undergoing RRP [21].

Minimally-invasive radical prostatectomy (MIRP) can also be offered to the obese patient, though the surgeon must consider the challenges unique to laparoscopic surgery. Both LRP and RALP require steep Trendelenburg positioning, and the attendant risks associated with transperitoneal or extraperitoneal laparoscopy. We review the outcomes of LRP and RALP in the obese patient. 

The surgical outcomes associated with LRP in the obese population are described in a number of series. In a review of conversions from LRP to open RRP, Bhayani found that obesity was a risk factor for conversion [7]. The authors suggest that obese patients should not be included early in a surgeon's LRP experience. Several authors have found that LRP operative time is increased in the obese population. Brown noted that operative time was increased by a mean of 29 minutes in patients with Class II and III obesity [7]. In this series, length of stay, complication rates, and pathological findings were similar in the obese and non-obese groups. Another series found similar results, with obesity adding an average of 38 minutes to the operative time [31]. Another group reported longer operative time by an average of 15 minutes, but similar estimated blood loss, length of stay, and foley catheter duration. [23] Interestingly, in this series, the PSM rate in obese patients with cT1c disease was twice that of similar non-obese patients, though this did not reach statistical significance. Erectile function was lower in the obese group, but again was not statistically significant. Similar findings were reported in a group of patients undergoing extraperitoneal LRP. Operative time in the obese patients averaged 20 minutes longer than the non-obese patients. The complication rates, foley catheter duration, urinary and erectile function at 6 month followup, and PSM were similar between obese and non-obese patients.

Robot-assisted laparoscopic prostatectomy has been described in obese patients, with outcomes similar to those of LRP. Mikhail et al. [14] noted similar conversion rates, complication rates, pathological stage, PSM rates, and sexual and urinary function in obese and non-obese patients. However, the first three conversions to open surgery occurred in obese patients due to bleeding, slow progression, or to optimize oncologic outcomes. The authors suggest that RALP can be safely performed in the obese patient once the surgeon is experienced with the technique. The authors also found that operative time and EBL was significantly greater in obese patients compared to non-obese patients. In an update from the same institution, Wiltz et al. [16] reports that EBL was similar between obese and non-obese patients, perhaps an improvement reflecting more experience. Several other interesting findings were noted. First, there was a higher open conversion rate among obese patients. Five of the six conversions in obese patients occurred during the initial 100 cases, and were due to failure of the surgery to progress appropriately. Overall, the case abortion rate was similar between obese and non-obese patients, but the incidence of aborted procedures due to respiratory distress was significantly higher in the obese patients. Obese patients also had lower preoperative Sexual Health Inventory for Men (SHIM) scores at baseline and less favorable return of continence and potency at 12 and 24 months compared to non-obese patients. The findings of this study suggest that a large series of patients with followup for greater than one year is ideal to detect differences between the obese and non-obese patient groups.

Ahlering also found that postoperative continence rates are worse in obese patients [6]. These patients also exhibited significantly lower baseline (preoperative) SHIM scores, peak urinary flow rates, urinary bother scores, and voided urinary volumes. The authors also found that obese patients experienced an increase in perioperative complications, and that these complications were more severe than those in non-obese patients.

Several other series report RALP outcomes in obese patients. Similar to other authors, Herman et al. [18] reported increased operative time and EBL among obese patients undergoing RALP. The obese patients also had a higher rate of PSM's, though there were no differences in pathological stage, Gleason score, complication rates, and the incidence of biochemical recurrence one year after surgery. Castle also noted greater operative time and EBL in the obese patients undergoing RALP [10]. In addition, this group found higher PSM rates and higher pathological tumor volumes in the obese patients. Boorjian also found a trend toward higher rates of PSM in the obese patients [8]. When evaluating the site of the PSM's, obese patients were more likely to have a posterior PSM, while non-obese patients more often had a PSM at the apex. The obese patients in this institution's series had greater EBL and operative time, but the length of stay, transfusion rates, and complication rates were similar. Finally, Boczko found that obese patients undergoing extraperitoneal RALP experienced similar operative times, EBL, preoperative PSA, clinical and pathological stages, specimen weights, PSM rates, 6 month continence rates, and complication rates when compared to non-obese patients [17]. 

Reported outcomes in patients undergoing MIRP vary between series. It is likely that a number of factors influence these outcomes. For instance, one of the institutions reported different results with a larger cohort of patients and longer followup. In addition, the degree of obesity varied between institutions and series, which undoubtably influence outcomes. 

We report a series of morbidly obese patients (BMI ≥ 40), with favorable perioperative outcomes, specifically no conversions to open surgery, case abortions, or early perioperative complications. These outcomes may be partly attributed to the robotic case volume at our institution, which allows the entire surgical team, including the operating room staff, anesthetist, and surgeon, to feel more comfortable with more challenging patients. 

 RALP in the morbidly obese patient can be challenging. Close communication with the anesthesiology team is critical, as these patients may be more sensitive to pneumoperitoneum and steep Trendelenburg. Cardiology clearance in these patients, who are more likely to have medical comorbidities [16], is well advised. When positioning the patient, the extremities must be carefully padded to avoid neuropraxia. The patient's weight should be taken into consideration to avoid any additional pressure on the arms when tucked at the patient's side. The table armboards may be added to provide additional room for adequate padding of the upper extremities. Trocar placement should be modified based on the patient's body habitus. Longer instruments and trocars may be necessary, and the trocars may actually need to be placed in a more cephalad position on the abdomen as measured from the pubic symphysis. This is necessary due to a more vertical trajectory from the skin toward the pelvis due to the protuberance of the abdomen [14]. Finally, RALP in the obese patient, and especially in the morbidly obese patient, is more likely to have favorable outcomes when performed later in the surgeon's learning curve. Once the technique of RALP has been mastered, and the surgeon can troubleshoot problems in the non-obese patient, the challenges posed by the obese patient can be more successfully overcome.

## 5. Conclusion

To our knowledge, this is the largest series reporting outcomes of morbidly obese patients undergoing RALP. We report an acceptable PSM rate, no conversions to open surgery, no case abortions, and few perioperative complications. Robot-assisted laparoscopic prostatectomy, while technically challenging in the obese patient, is an appropriate surgical option when offered by experienced surgeons. 

## References

[1] Hedley AA, Ogden CL, Johnson CL, Carroll MD, Curtin LR, Flegal KM (2004). Prevalence of overweight and obesity among US children, adolescents, and adults, 1999–2002. *Journal of the American Medical Association*.

[2] Calle EE, Teras LR, Thun MJ (2005). Obesity and mortality. *The New England Journal of Medicine*.

[3] Siddiqui SA, Inman BA, Sengupta S (2006). Obesity and survivial after radical prostatectomy: a 10-year prospective cohort study. *Cancer*.

[4] Freedland SJ, Grubb KA, Yiu SK (2005). Obesity and risk of biochemical progression following radical prostatectomy at a Tertiary Care Referral Center. *Journal of Urology*.

[5] Jayachandran J, Bañez LL, Aronson WJ (2009). Obesity as a predictor of adverse outcome across black and white race: results from the shared equal access regional cancer hospital (SEARCH) database. *Cancer*.

[6] Ahlering TE, Eichel L, Edwards R, Skarecky DW (2005). Impact of obesity on clinical outcomes in robotic prostatectomy. *Urology*.

[7] Bhayani SB, Pavlovich CP, Strup SE (2004). Laparoscopic radical prostatectomy: a multi-institutional study of conversion to open surgery. *Urology*.

[8] Boorjian SA, Crispen PL, Carlson RE (2008). Impact of obesity on clinicopathologic outcomes after robot-assisted laparoscopic prostatectomy. *Journal of Endourology*.

[9] Brown JA, Rodin DM, Lee B, Dahl DM (2005). Laparoscopic radical prostatectomy and body mass index: an assessment of 151 sequential cases. *Journal of Urology*.

[10] Castle EP, Atug F, Woods M, Thomas R, Davis R (2008). Impact of body mass index on outcomes after robot assisted radical prostatectomy. *World Journal of Urology*.

[11] Khaira HS, Bruyere F, O’Malley PJ, Peters JS, Costello AJ (2006). Does obesity influence the operative course or complications of robot-assisted laparoscopic prostatectomy. *BJU International*.

[12] Liatsikos E, Mühlstädt S, Kallidonis P (2008). Performance and functional outcome of endoscopic extraperitoneal radical prostatectomy in relation to obesity: an assessment of 500 patients. *BJU International*.

[13] Lindner U, Lawrentschuk N, Abouassaly R, Fleshner NE, Trachtenberg J (2010). Radical prostatectomy in obese patients: improved surgical outcomes in recent years. *International Journal of Urology*.

[14] Mikhail AA, Stockton BR, Orvieto MA (2006). Robotic-assisted laparoscopic prostatectomy in overweight and obese patients. *Urology*.

[15] Singh A, Fagin R, Shah G, Shekarriz B (2005). Impact of prostate size and body mass index on perioperative morbidity after laparoscopic radical prostatectomy. *Journal of Urology*.

[16] Wiltz AL, Shikanov S, Eggener SE (2009). Robotic radical prostatectomy in overweight and obese patients: oncological and validated-functional outcomes. *Urology*.

[17] Boczko J, Madeb R, Golijanin D (2006). Robot-assisted radical prostatectomy in obese patients. *The Canadian Journal of Urology*.

[18] Herman MP, Raman JD, Dong S, Samadi D, Scherr DS (2007). Increasing body mass index negatively impacts outcomes following robotic radical prostatectomy. *JSLS*.

[19] Yang BK, Gan TJ, Salmen CR, Cancel QV, Vieweg J, Dahm P (2006). Radical perineal prostatectomy for treatment of localized prostate cancer in obese and nonobese patients: a matched control study. *Urology*.

[20] Jayachandran J, Aronson WJ, Terris MK (2008). Obesity and positive surgical margins by anatomic location after radical prostatectomy: results from the shared equal access regional cancer hospital database. *BJU International*.

[21] van Roermund JGH, van Basten J-PA, Kiemeney LA, Karthaus HFM, Witjes JA (2009). Impact of obesity on surgical outcomes following open radical prostatectomy. *Urologia Internationalis*.

[22] Boczko J, Melman A (2003). Radical perineal prostatectomy in obese patients. *Urology*.

[23] Eden CG, Chang CM, Gianduzzo T, Moon DA (2006). The impact of obesity on laparoscopic radical prostatectomy. *BJU International*.

[24] Meininger D, Zwissler B, Byhahn C, Probst M, Westphal K, Bremerich DH (2006). Impact of overweight and pneumoperitoneum on hemodynamics and oxygenation during prolonged laparoscopic surgery. *World Journal of Surgery*.

[25] Choban PS, Flancbaum L (1997). The impact of obesity on surgical outcomes: a review. *Journal of the American College of Surgeons*.

[26] Dindo D, Muller MK, Weber M, Clavien P-A (2003). Obesity in general elective surgery. *The Lancet*.

[27] Hollenbeak CS, Rogers AM, Barrus B, Wadiwala I, Cooney RN (2008). Surgical volume impacts bariatric surgery mortality: a case for centers of excellence. *Surgery*.

[28] Ballantyne GH, Belsley S, Stephens D (2008). Bariatric surgery: low mortality at a high-volume center. *Obesity Surgery*.

[29] Bolenz C, Gupta A, Hotze T (2010). The influence of body mass index on the cost of radical prostatectomy for prostate cancer. *BJU International*.

[30] Freedland SJ, Isaacs WB, Mangold LA (2005). Stronger association between obesity and biochemical progression after radical prostatectomy among men treated in the last 10 years. *Clinical Cancer Research*.

[31] El-Feel A, Davis JW, Deger S (2003). Laparoscopic radical prostatectomy—an analysis of factors affecting operating time. *Urology*.

